# One-year outcome after repair of giant incisional hernia using synthetic mesh or full-thickness skin graft: a randomised controlled trial

**DOI:** 10.1007/s10029-019-01900-4

**Published:** 2019-02-08

**Authors:** V. Holmdahl, B. Stark, L. Clay, U. Gunnarsson, K. Strigård

**Affiliations:** 1 Department of Surgery and Perioperative Sciences, Umeå University, Daniel Naezéns väg, 90185 Umeå, Sweden; 2 Department of Molecular Medicine and Surgery, Karolinska Institute, Solna, Sweden; 3 Department of Clinical Science and Education, Karolinska Institute, Solna, Sweden

**Keywords:** Incisional hernia, Ventral hernia, Full-thickness skin, Recurrence rate, Abdominal muscle strength

## Abstract

**Purpose:**

Repair of giant incisional hernia often requires complex surgery and the results of conventional methods using synthetic mesh as reinforcement are unsatisfactory, with high recurrence and complication rates. Our hypothesis was that full-thickness skin graft (FTSG) provides an alternative reinforcement material for giant incisional hernia repair and that outcome is improved. The aim of this study was to compare FTSG with conventional materials currently used as reinforcement in the repair of giant incisional hernia.

**Methods:**

A prospective randomised controlled trial was conducted, comparing FTSG with synthetic mesh as reinforcement in the repair of giant (> 10 cm minimum width) incisional hernia. One-year follow-up included a blinded clinical examination by a surgeon and objective measurements of abdominal muscle strength using the Biodex-4 system.

**Results:**

52 patients were enrolled in the study: 24 received FTSG and 28 synthetic mesh. Four recurrences (7.7%) were found at 1-year follow-up, two in each group. There were no significant differences regarding pain, patient satisfaction or aesthetic outcome between the groups. Strength in the abdominal wall was not generally improved in the study population and there was no significant difference between the groups.

**Conclusion:**

The outcome of repair of giant incisional hernia using FTSG as reinforcement is comparable with repair using synthetic mesh. This suggests that FTSG may have a future place in giant incisional hernia repair.

## Introduction

Incisional hernia is a common complication of abdominal surgery, often causing considerable suffering for the patient [1]. The degree of symptoms is related to the size of the hernia and there is a strong inverse correlation between abdominal wall muscle strength and the area of the hernia [2, 3].

Several complex factors must be taken into consideration when planning surgical reconstruction of a giant incisional hernia. The complexity is partly due to the mere size of the hernia where approximation of fascial borders can require the use of special techniques such as component separation [4]. Another factor is loss-of-domain, i.e. a significant proportion of abdominal contents being displaced from the abdominal cavity into the hernia sac. Reinsertion of the contents at surgery can lead to respiratory problems [2, 5]. Patients with a giant hernia may also have a biologic predisposition to hernia development due to an imbalance in tissue and serum matrix metalloproteinases and their inhibitors [6].

Repair using synthetic mesh material for reinforcement, as used for less complex hernia repairs with low recurrence rates, is unsatisfactory in giant incisional hernia repair where recurrence rates can exceed 30% and complications are more common and more serious [2, 5, 7–9]. Furthermore, infection related to the use of synthetic material, as well as long-term complaints such as chronic pain, dysfunction of the abdominal wall, enterocutaneous fistulae and discomfort, is seen [5, 10]. An alternative reinforcement material providing lower recurrence rates, better comfort and fewer side effects is thus highly desirable. Due to their extremely high cost, currently available biological prosthetic materials are mainly used as a last resort in cases such as repair in a contaminated surgical field, and results regarding recurrence have not lived up to expectations [11].

Our hypothesis was that the use of autologous full-thickness skin graft (FTSG) is an alternative reinforcement material that improves outcome. The use of FTSG as reinforcement in hernia repair was introduced in the early twentieth century and was followed by an increasing interest during the first half of the twentieth century because of encouraging results [12–15]. Histological studies have shown that subcutaneous implantation of FTSG does not give rise to cyst formation or malignant change, and recent animal studies have shown excellent graft survival in the abdominal wall [16, 17]. However, interest in this technique faded with the introduction of synthetic materials such as polypropylene and polyester, since when FTSG has only been used sporadically in selected cases [18, 19]. Because of the increasing awareness of the problems related to the use of current synthetic materials, it is time to reassess alternative methods such as FTSG [5, 10].

This study was a 1-year follow-up of a randomised controlled trial comparing FTSG to synthetic mesh in surgical repair of giant incisional hernia. A 3-month follow-up with early surgical complication as the primary outcome has recently been reported [20]. In this 1-year follow-up of the same patient cohort, we investigated the following outcomes: hernia recurrence, pain, abdominal wall discomfort, experienced improvement, and abdominal wall muscle strength measured by the Biodex system.

## Materials and methods

### Patients

Patients with symptomatic giant (in this study defined as > 10 cm minimum width) ventral incisional hernia were selected for the study. Exclusion criteria were under 18 years of age, ongoing immunosuppressive treatment, ongoing smoking, and ongoing pregnancy or nursing. These criteria were chosen because of their potential contribution to the risk for postoperative respiratory problems and impaired wound healing [20].

### Study design

A prospective randomised controlled trial was carried out at two Swedish university hospitals specialised in advanced abdominal wall and hernia surgery. A power calculation was not performed for the endpoints investigated in the present study but was made for the 3-month complication rate study on the same material. In that study, the power calculation showed that a sample of 50 patients was required to achieve 80% power and 95% significance with estimated short-term surgical complication rates of 50% in the synthetic group and 20% in the FTSG group [20]. Randomisation was accomplished by a research nurse using unmarked envelopes containing a sheet of paper bearing the name of one of the two study methods. The envelopes were opened 1 day prior to surgery and the method to which the patient was allocated was revealed to the surgical team only. Further details on the randomisation process have been described elsewhere [20].

### Preoperative preparation

Patients in both groups underwent computerised tomography scan (CT scan) of the abdominal wall. An objective way to measure the effect of a giant hernia and its repair on daily activities is to assess abdominal wall muscle strength using the Biodex system™ (Biodex Corp. Shirley, NY, USA) [21]. The Biodex system has been validated and shown to be reliable in patients with giant ventral hernia as well as abdominal rectus diastasis [22, 23]. Improvement in core strength has been confirmed after surgical repair of ventral hernia [24, 25]. All patients included in the study had their abdominal muscle strength measured in the sitting position using the Biodex multi-joint system-4. Five different strength modalities were measured: peak isometric torque, peak torques of flexion and extension at speed 30°/s, and the same at 60°/s. Two specially trained physiotherapists, blinded to the surgical method allocated, followed a standard protocol including patient instructions, and the same procedure was performed at follow-up.

### Surgical procedure

The primary surgeon at all operations was one of two senior surgeons with a vast experience of abdominal wall surgery. The synthetic mesh used was a polypropylene mesh overlapping the repaired hernia defect by a minimum of 5 cm. The aim was to place the mesh in the retromuscular space, but onlay and IPOM positions were not excluded if deemed necessary. The FTSG was always placed in an onlay position, also aiming to obtain an overlap of > 5 cm. The FTSG was in all cases taken from excess skin adjacent to the midline incision made for the hernia repair. A senior plastic surgeon took part in most FTSG procedures. Details of the surgical procedures employed have been described in a previous publication [20]. At the initiation of the study, there was not enough support in the literature to place the skin graft safely in any other position than onlay and that this position had been tested in a proof-of-concept study [26].

### Postoperative management

Postoperative care was identical in both groups, with nursing staff and patients blinded to the procedure performed, throughout the study period. Early mobilisation was practised and patients in both groups were provided with an elastic girdle and instructed to wear it day and night for the first 6 weeks followed by daytime use for a further 6 weeks.

### Study outcomes

After 1 year, all patients were scheduled for follow-up with a repeat Biodex investigation and a clinical examination focusing on signs of recurrence. If recurrence could not be ruled out by clinical examination, a new CT scan was performed. The radiologist examining the CT scans was blinded to the surgical technique used. The aesthetic outcome, i.e. appearance of the scar area regarding uneven distribution or excess skin, and adequacy of wound healing, was also evaluated. Pain and overall improvement in preoperative complaints were assessed using the questions “Do you experience pain from the abdominal wall?” and “Do you experience an improvement in abdominal wall function?” The patient described their pain or improvement using a visual analogue scale (VAS) from zero (least) to ten (most). Follow-up assessment was performed by an experienced surgeon blinded to the surgical procedure and not involved in the study.

### Statistics

All data were gathered in an Access™ database (Microsoft, Redmond, Washington, USA). The area of the hernia was regarded as an ellipse. Statistical analyses were carried out on SPSS 24® (IBM corp., Armonk, New York, USA). Mean comparisons for continuous variables were carried out using independent- and paired *t* tests, and the Mann–Whitney *U* test for non-parametric variables. Dichotomous variables were tested using Chi-square statistics and Fisher’s exact test when Chi-square criteria were not met. A *p* value < 0.05 was considered significant. All patients randomised were analysed according to the intention-to-treat principle. Linear regression analysis was used to investigate correlations between baseline characteristics and Biodex measurement outcome. All variables were simultaneously entered in the multivariate analysis.

## Results

**Fig. 1:**
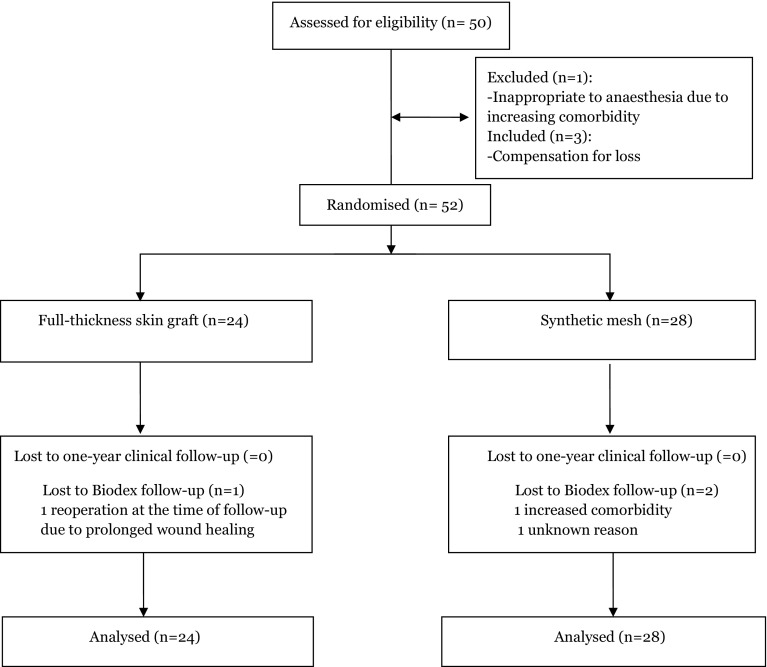
Consort 2010 flow diagram

All patients were recruited from December 2009 until August 2013. After 50 patients had been assessed for eligibility, one was excluded due to cancellation of surgery due to such an increase in comorbidity that anaesthesia was deemed inappropriate. To compensate for this exclusion, three more patients were enrolled in the study (Fig. 1). Baseline characteristics of the groups are presented in Table 1. No significant differences were seen between the groups.

**Table 1:** Baseline

	FTSG (*n* = 24)	Synthetic (*n* = 28)	*p* value
Age	64 (7.8)	64 (14.8)	0.847
BMI	31.2 (8.2)	31.0 (9.8)	0.741
Gender (male/female)	12/12	15/13	0.797
Peroperative area of hernia	137.4 (104.3)	150.8 (170.4)	0.400

Median (Interquartile range)

Preoperative baseline data. Age in years

BMI in kg/m^2^

Numbers of males and females, respectively, are shown under gender

Area of hernia in cm^2^

All patients were treated with the intended surgical procedure.

Table 2 shows data from the 1-year follow-up. There were four recurrences (7.7%) with no significant difference between the groups. One recurrence in each group was already evident at the 2-month follow-up. The majority of the study population experienced considerable subjective improvement in abdominal wall function; the FTSG-group having a median of eight and the synthetic group six on a ten-grade VAS, with no significant difference between the groups. In general, no pain was perceived by the study population at follow-up. A considerable number of patients had excess skin and/or uneven distribution of skin over the abdomen, but there was no significant difference between groups.

**Table 2:** One-year follow-up

	FTSG	*n*	Synthetic	*n*	*p* value
Recurrence	2 (8.3%)	24	2 (7.1%)	28	1.000**
Well-healed scar	21 (91.3%)	23	27 (96.4%)	28	0.439
Excess skin	14 (60.9%)	23	16 (57.1%)	28	0.788
Uneven distribution	12 (52.2%)	23	15 (53.6%)	28	0.921
Experienced improvement	8 (4)*	22	6 (7)*	28	0.074
Pain	0 (2)*	24	0 (5)*	28	0.201

Clinical examination of the surgical site at 1-year follow-up. Numbers of patients with occurrence of the outcome are presented, percentage of the group in parentheses. Experienced improvement and pain represent the VAS answer to the questions “Do you experience an improvement of the abdominal wall function?” and “Do you experience pain from the abdominal wall?” *Median (interquartile range). **Fisher’s exact test

**Fig. 2:**
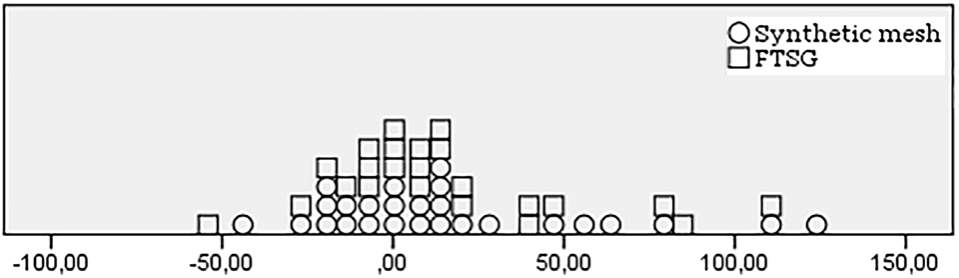
Percentage change in Biodex for all modalities combined. Each dot represents an individual

In general, no significant improvement in abdominal muscle strength measured with the Biodex-4 was seen after surgery, regardless of technique (Table 3). However, there was significant inter-individual variability as seen in Fig. 2, where the majority of patients experienced some degree of improvement.

**Table 3:** Biodex at 1-year follow-up

	Synthetic		FTSG		*p* value*	Total population		*p* value**
	Mean	Standard deviation (Nm)	Mean	Standard deviation (Nm)		Mean	Standard deviation (Nm)	
Flex 30	11.18 (29%)	36.43	3.05 (16%)	35.71	0.535	7.37 (23%)	35.95	0.158
Flex 60	4.45 (12%)	42.32	− 2.69 (10%)	42.11	0.782	0.95 (11%)	41.91	0.877
Ext 30	5.60 (13%)	36.51	5.10 (19%)	36.34	0.616	5.36 (16%)	36.05	0.303
Ext 60	8.48 (13%)	45.37	− 1.73 (7%)	35.77	0.733	3.69 (10%)	41.05	0.532
Isometric	4.49 (17%)	19.92	2.71 (28%)	18.76	0.812	3.62 (22%)	19.16	0.212
Total	6.88 (16%)	32.17	1.40 (16%)	25.15	0.514	4.31 (16%)	28.92	0.275

Mean change in Biodex at 1-year follow-up in Nm, mean percentage change in parentheses. In the bottom row, the mean of all exercises is presented. *Independent *t* test, **paired *t* test

No significant relationship between baseline characteristics and the difference in abdominal muscle strength before and after surgery was seen in the univariate linear regression model or the multivariate (Table 4).

**Table 4:** Linear regression analysis

	Univariate				Multivariate			
	Β-Coefficient	95% confidence interval		*p* value	Β-Coefficient	95% confidence interval		*p* value
Age	− 0.471	− 1.378	0.437	0.302	− 0.430	− 1.378	0.517	0.365
Area of hernia	0.058	− 0.024	0.139	0.161	0.055	− 0.029	0.140	0.194
BMI	0.250	− 1.214	1.714	0.733	0.071	− 1.539	1.682	0.929
Female sex (vs male)	5.367	− 11.362	22.095	0.522	4.168	− 13.890	22.226	0.644
Synthetic mesh (vs FTSG)	5.479	− 11.275	22.233	0.514	5.163	− 11.803	22.129	0.543

Linear regression analysis on how different baseline characteristics influence average change in Biodex. Same units on explanatory variables as in Table 1

*FTSG* full-thickness skin graft

## Discussion

The results in this study indicate that there is little difference between using conventional synthetic mesh and FTSG in the repair of giant incisional hernia. The overall 1-year recurrence rate in this study was low, with no significant differences between FTSG and synthetic mesh as reinforcement [2, 5]. Likewise, there were no differences in outcome regarding pain, aesthetic results and subjective improvement between mesh and FTSG.

These results together with previous studies on short-term surgical complications on the same cohort of patients pave the way for further trials using FTSG as an alternative to conventional synthetic mesh for reinforcement in the repair of giant incisional hernia. An interesting aspect would be to evaluate the potential benefits of using autologous tissue in patients with immunosuppressive treatments and conditions, as well as which method that is most appropriate in contaminated conditions. Since all FTSG could be taken adjacent to the midline incision, the potential morbidity of an additional incision could be eliminated in this study, which otherwise could constitute a drawback with the FTSG-method. Furthermore, since FTSG offers many of the advantages of currently available biologic mesh materials but without the immunological response, it has the potential to offer better long-term results. However, long-term follow-up is necessary to reveal potential late recurrences and complications that are not evident at 1-year postoperatively, and a long-term follow-up is also included in the study protocol for this RCT. Another advantage is that the graft material is “free of charge”, so the FTSG method could reduce costs considerably in this patient group. Cost-effectiveness, however, was not considered in this study.

In contrast to previous studies, giant incisional hernia repair did not improve abdominal wall muscle strength measured by the Biodex system at the 1-year follow-up [24, 25]. Moreover, there was no difference between groups using synthetic mesh or FTSG as reinforcement material. Placement of reinforcement material in the abdominal wall was chosen pragmatically to achieve best possible results with safety maintained, and this resulted in different positioning between groups. However, a previous study found that placement of the reinforcement mesh in the abdominal wall does not affect abdominal muscle strength [27].

The relatively large variability in improvement in abdominal muscle strength compared to preoperative values suggests the need to investigate which individuals stand to gain the greatest improvement in strength from surgery; unfortunately, regression analysis in this study gave no clues to possible indicators. One reason for the absence of overall improvement in abdominal muscle strength may be that some of these patients had had their hernia a long time. Long-term imbalance in distribution of forces in the abdominal wall gives rise to irreversible atrophy of muscles and other tissues as shown in rat models by Dubay et al. [28]. This may have led to permanent weakening of the abdominal wall muscles in some individuals in our cohort of patients with giant ventral hernia. The importance of the age of the hernia is further emphasised by the fact that there is a positive correlation between size of hernia and abdominal wall muscle strength [3]. Pre- and postoperative physiotherapy as well as prolonged active rehabilitation may be a necessary complement to surgery if one is to recover muscle strength.

It is important to understand that incisional hernia is a condition that becomes increasingly difficult to manage with time, and should, therefore, be given priority to avoid it becoming a giant hernia with associated complications [9].

Another complicating factor regarding repair of giant hernia is loss-of-domain. Reinsertion of a large volume of extra-abdominal bowel is a significant risk factor for respiratory problems, which in turn limits the possibility of adequate rehabilitation. There is no gold standard for the accurate assessment of loss-of-domain and how likely it is to affect the results of surgery. In the present study, patients were included regardless of the degree of loss-of-domain, which could explain the large variability in Biodex results.

Giant incisional hernia is the result of failure to heal after previous surgery. In this study, we did not consider previous underlying complications such as infection and poor surgical technique. Because of the very nature of incisional hernia, it tends to afflict a group of patients prone to develop hernia because of biological abnormalities. In a recent study on the biochemistry of abdominal wall tissue, disturbances in the expression of matrix metalloproteinases and distribution of collagen were shown to influence the risk for developing hernia [6].

One strength of this study is that the Biodex system has been validated for giant ventral hernia and is the most extensively used measurement technique for assessing abdominal wall strength [22, 29]. It not only gives a simple assessment of flexor strength but also provides an overall picture of abdomino-lumbar girdle function in its entirety. Another strength is the fact that the patients, nursing staff, physiotherapists and postoperative surgical evaluators were blinded to the randomisation preventing investigator or patient bias.

The sample size of the groups was calculated for a study on early surgical complications, and we cannot exclude the possibility that the same groups were too small to reveal significant differences for other parameters. Another limitation of this study is that it did not investigate in detail any difference in quality-of-life after the procedures. The secondary outcome “experienced improvement*”* gave only a limited insight into the general feeling of well-being the patients experienced. A 3-year follow-up focusing on various aspects of quality-of-life is, therefore, planned.

## Conclusion

This study showed no significant difference in recurrence rates when using FTSG or conventional synthetic mesh as reinforcement material in giant incisional hernia repair. Objective measurement of abdominal wall muscle strength 1 year after surgery did not reveal any significant improvement compared to preoperative values, and no difference was seen between the two groups. The similarity in results between these techniques indicates that FTSG possibly has a future role in hernia repair, but more research is needed.
